# Wave Equation Modeling via Physics-Informed Neural Networks: Models of Soft and Hard Constraints for Initial and Boundary Conditions

**DOI:** 10.3390/s23052792

**Published:** 2023-03-03

**Authors:** Shaikhah Alkhadhr, Mohamed Almekkawy

**Affiliations:** 1 School of Electrical Engineering and Computer Science, Pennsylvania State University, University Park, PA 16802, USA; 2 Information Science Department, Sabah AlSalem University City, Kuwait University, P.O. Box 25944, Safat 1320, Kuwait

**Keywords:** numerical modeling, physics-informed neural networks, wave equation, ultrasound therapeutics

## Abstract

Therapeutic ultrasound waves are the main instruments used in many noninvasive clinical procedures. They are continuously transforming medical treatments through mechanical and thermal effects. To allow for effective and safe delivery of ultrasound waves, numerical modeling methods such as the Finite Difference Method (FDM) and the Finite Element Method (FEM) are used. However, modeling the acoustic wave equation can result in several computational complications. In this work, we study the accuracy of using Physics-Informed Neural Networks (PINNs) to solve the wave equation when applying different combinations of initial and boundary conditions (ICs and BCs) constraints. By exploiting the mesh-free nature of PINNs and their prediction speed, we specifically model the wave equation with a continuous time-dependent point source function. Four main models are designed and studied to monitor the effects of soft or hard constraints on the prediction accuracy and performance. The predicted solutions in all the models were compared to an FDM solution for prediction error estimation. The trials of this work reveal that the wave equation modeled by a PINN with soft IC and BC (soft–soft) constraints reflects the lowest prediction error among the four combinations of constraints.

## 1. Introduction

Partial differential equations (PDEs) are some of the most significant challenges in the field of scientific computing, and they have been rigorously approached using different methods [1]. Numerical modeling is a powerful mathematical tool in medical, industrial, and academic fields [2]. Regardless of the field of application, it is essential to understand the system. Modeling a particular system can provide a clear view of its momentous components and affecting factors. Thus, it unlocks development, control, and maintenance insights [3].

In particular, numerical methods have a specific significance in modeling ultrasound waves, characterizing the acoustic field, designing ultrasound transducers, and ultrasound treatment planning [4,5,6]. Studying the physical nature of acoustics, especially in ultrasound therapeutics, represents a substantial contribution to noninvasive medical procedures. The ability to simulate the propagation of ultrasound waves within a domain in the human body has an extensive impact on confidence and success prior to the initiation of therapy. This reduces the possibility of erroneous setups, validates safety factors, reduces treatment planning and patient waiting times, and eventually reduces the overall cost of the medical procedure [4,7,8].

Partial differential wave equations are traditionally modeled using tools such as the Finite Difference Method (FDM) [9,10], Finite Element Method (FEM) [11,12], or spectral methods [13]. These methods typically rely on polynomials, piecewise polynomials, and other basic functions. Given the methodology of these approaches, the modeled problem must be set up on a mesh (grid) of finite points. Although they are considered elegant and practical strategies, their applicability is easily hindered as the number of dimensions increases. Owing to their mesh-based nature, the increase in dimensions is paired with an increase in computational operations and resource allocation. This modeling complication is referred to as the Curse of Dimensionality (CoD) [14]. This is one of the most common obstacles in PDE modeling. Another concern that accompanies mesh-based approaches is discretization. The PDE was discretized and then solved through time-stepping. The discretization error when the grid size is not sufficiently small to capture the desired resolution of the modeled system can reflect incorrect results [15]. The term “traditional methods” here also covers methods that provide a solution in a converging series form, such as the Taylor series [16]. This steers the process of solving PDEs into other lanes of complexity, as the solution may require multiple series terms to ensure minimal error and quick convergence.

Continuous research on artificial intelligence, along with advancements in computing power, has spawned a new field of modeling techniques utilizing Deep Learning (DL) [17,18]. Neural Networks (NNs) have been considered universal PDE modeling tools for an extended period of time that stretches back to the 1990s [19]. One popular option for solving forward problems is Deep Neural Networks (DNNs), where they are trained to predict the solution to a defined physics problem [20,21,22]. Despite their potential, being a data-driven approach requires a relatively large number of training datasets. Sufficient training datasets are commonly lacking for many specific problems. In addition, the DNN training process can be challenging because of the difficulty in determining the optimal hyperparameters for the NN.

A recently introduced class of DNNs was explicitly used for solving PDEs by exploiting the physics of the problem. This class of DNNs is referred to as Physics-Informed Neural Networks (PINNs) [23]. Unlike the normal DNN, which requires a previous solution of the PDE to perform training with input and output pairs, PINNs account for the physical concept of the problem by incorporating the formula of the governing physics law along with its initial and boundary conditions into the loss function. The PINN is then trained to minimize the loss value. During the training iterations, PINN efficiently employs the feature of automatic differentiation to compute partial derivative terms with respect to space or time. Therefore, PINNs are a mesh-free approach [24,25,26,27].

PINNs can overcome the CoD problem faced in traditional modeling methods by predicting the PDE solution without the need to construct detailed grids. A few differences between PINNs and traditional methods are highlighted here. Instead of using a mesh for spatiotemporal stepping, PINNs rely on irregularly sampled points from the defined domain via different sampling distributions [28]. To approximate the PDE solution, PINNs use the nonlinear representation of the NN instead of the linear piecewise polynomials used in traditional methods. The parameters to be optimized in PINNs are the NN weights and biases, whereas in traditional methods, the optimization focus is on the point values on the formed grid. The PDE is embedded in the form of a loss function in PINNs instead of as an algebraic matrix (system) in traditional methods. In addition, gradient optimizers [29] are error minimizers in PINNs, in contrast to the linear solvers in traditional methods.

Using the location of domain points as the training set, PINNs have the distinctive feature of not requiring a previously computed solution for the training process. However, like any other NN used for modeling forward problems, the training process can be a strenuous task for problems exhibiting high-frequency or multiscale features. Designing PINNs, although conceptually simple, requires significant trial and testing to determine the best PINN model for the PDE problem, especially because PINN models are highly problem-dependent [28].

The wave equation has been modeled by PINNs previously in [30], showing the possibility of accurately modeling the wave equation in 2D and in an inhomogeneous domain. However, the PINN model conditions the initial state as a single-pulse source point. Hence, the model focused on the propagation of a single wave rather than a continuous or periodic time-dependent wave source. The wave equation was also solved with a PINN-based model to complement the limited available data in [31], where, similar to the previous approach, the source was implemented as a perturbation captured in the initial condition. This implementation of the wave source is simpler than our focus on periodically generating waves from a time-dependent function. In ref. [32], the wave equation was modeled using PINNs and compared to the solution of the Gaussian Process (GP). The focus of that work was mainly on exploring the accuracy and noise tolerance of the two approaches instead of the setup of the problem constraints.

PINN has also been used to solve the wave equation in the frequency domain [33]; assuming the wave is propagating in an infinite domain, the PINN-based model did not use boundary conditions. Therefore, no particular significance has been dedicated to establishing this condition. In ref. [34], the effects of enlarging the PINN architecture and increasing randomly selected training points on the loss value were discussed. In that work, an extension to the PINN architecture was implemented to correspond to a perfectly matched layer at the boundaries, which reportedly increases the cost of training. The PINN model studied there has demonstrated reasonable predictions for the real and imaginary parts of the wavefield in different media. However, the solution is studied in the frequency domain, and no particular attention was dedicated to the implementation of the constraints’ statuses.

In the observed previous literature on using PINNs to solve the wave equation, albeit using different successful approaches of this tool to model wave propagation, there is still a lack of specialized studies on the best PINN IC and BC constraint implementations for the wave equation. Moreover, the available literature has yet to touch on implementations of the continuous (periodically generated) wave from a time-dependent point source function for some ultrasound therapeutic applications.

Since the initial and boundary conditions of a PDE problem can be implemented in PINNs as soft or hard constraints, the primary question we would like to answer in this research is: *How can we achieve the most accurate prediction of the forward wave equation, given the options of soft or hard constraint implementations of the initial and boundary conditions (ICs and BCs)?* In this work, we introduce a comprehensive comparison of different combinations of soft and hard constraints to implement ICs and BCs in a homogeneous domain. The wavefield model considered a single sinusoidal time-dependent function as the source point. Each PINN prediction was compared to the FDM solution. A series of experiments was performed to compare the performance of PINNs using different constraint statuses while applying the most suitable tested hyperparameters for each experiment. We then provide the average L2 relative error values to compare each case with its peer constraint combinations. To the best of our knowledge, we propose the first study on the differences between using soft and hard constraints to implement the ICs and BCs of the wave equation. In addition, instead of using the common PINN implementation of the source point in the initial state of the problem as sin(2πx), we employ the boundary condition as a time-dependent point source function (i.e., sin(2πft)). Using the results in our work, we demonstrate the flexibility of using soft constraints, the forcing effect of hard constraints, their effects on the average error values, and the trade-offs of each.

For the remainder of this article, Section 2 presents the significance of the wave equation and PINN design for the wave equation forward model along with the studied constraint statuses. Section 3 exhibits the performance of using different constraint combinations when using PINNs and reveals the best constraint implementation. Section 4 presents concluding remarks and future directions of this work.

## 2. Materials and Methods

Modeling ultrasound waves requires an understanding of the physical nature of the waves and their propagation through a medium. In this section, we discuss the wave equation and provide an overview of the PINNs. We utilize the concept of physics-informed deep learning to address the challenges of modeling wave equations.

### 2.1. The Wave Equation Model

The noninvasiveness, portability, and affordability of medical ultrasound procedures in clinical applications have led to much research investment [4]. Ultrasound waves can be utilized for various purposes in medical ultrasounds [35]. However, the delivery of ultrasound waves through different regions of the human body can significantly affect wave transmission. To account for such challenges, numerical modeling methods have been extensively used to predict wave propagation in a medium. We discuss the physical significance and importance of the wave PDE. One of the most commonly studied second-order hyperbolic PDEs for ultrasound applications is the wave equation. Wave propagation, compression, and expansion, possibly through different regions of a medium, can create a complex wavefield domain for modelling [30]. This makes wave propagation a substantial area of interest in ultrasound therapeutics and imaging, as it aidsin procedure planning and execution [7,36,37]. Equation (1) shows the lossless 1D wave equation [38],

(1) $$\frac{\partial^{2}u\left(x,t\right)}{\partial t^{2}}=c^{2}\left(\frac{\partial^{2}u\left(x,t\right)}{\partial x^{2}}\right),$$

where u(x,t) denotes the displacement of point *x* at time instance *t*. We assume that *c* is a constant that represents the speed of sound in the medium [39]. The absence of nonlinear terms in this particular equation makes it the simplest form of wave equation [4]. Nevertheless, modeling such an equation is considered a computational challenge in high-dimensional representation because of the oscillatory and multi-scale tendency of its solution. To guarantee a unique solution to this second-order PDE, strict initial and boundary conditions must be defined while constructing the problem domain [30].

Generally, modeling the wave equation requires four main steps [37]. The first step is to define the geometry of the domain and its properties. In this work, we use a constant value of *c* over a spatial vector *x* and the temporal range *t* to represent a homogeneous one-dimensional domain. The second step was to determine the transducer (source) function and its position. In our setup, the source point is placed on the left boundary of the domain and assigned a time-dependent continuous sinusoidal function S(x,t) as shown in Equation (2),

(2) S(x,t)=sin(aπft),

where *a* is a constant value that affects the period of the wave and *f* is the frequency. The third step was to set the model parameters, including the initial and boundary conditions of the modeled domain, as shown in Equations (3) and (4). This is normally the step in which the mesh size is specified in traditional numerical modeling methods. As we use PINNs to model the wave equation, this is not considered a concern given the mesh-free nature of this approach. Finally, the results obtained after applying the numerical method are processed.

(3) $$f_{i}\left(x,t\right)=0$$

(4) $$f_{lb}\left(x=0,t\right)=S\left(x,t\right),\;f_{rb}\left(x=1,t\right)=0\;$$

### 2.2. Physics-Informed Neural Networks (PINNs)

PINNs are a class of DNNs that function as PDE approximation tools. Contrary to the default functionality of a regular NN, where it fits itself to the solution data pairs of state and value in a supervised training process, the general idea in PINN is to train the NN by considering the physics of the problem. This is achieved by involving physics, normally a PDE, in the loss function. The training process then minimizes this loss function. The data for training is a set of points randomly selected from a defined domain, such as a 1D line, a 2D rectangular area, or a higher-dimensional geometry. Consequently, once the training is completed, the PINN can predict the physical behavior of any given point within that domain throughout a defined time interval. During the training process, no previous solution to the PDE is required; however, PINN learns the solution of the PDE while attempting to minimize its loss value. Randomly selected points from the domain are irregularly sampled using a specified distribution scheme (Sobol, uniform, pseudo-random, etc. [40]). These points are referred to as the collocation points [41]. Given the boundary conditions and the initial state of that particular system, PINN predicts the estimated PDE solution at the collocation points within the defined spatial and temporal windows. The nomenclature of combining informative physics laws with deep learning as a concept can be recognized under multiple titles such as physics “-informed,” “-guided,” or “-constrained” NNs.

As previously stated, PINNs have the ability to solve a PDE without the need for prior solutions, linearization, or local time-stepping [23]. The main feature of PINNs is their ability to predict the solution of linear or nonlinear PDEs in a mesh-free setup, without the prerequisite of a known solution for training. This classifies PINNs as unsupervised learning methods for forward problems. Given that it requires a relatively low number of training points [28], it is also an efficient tool for modeling multidimensional domains [23], such as an ultrasound transducer that transmits waves into a domain in the human body. PINNs involve the physics of a system in their loss function. This forces the NN to consider the PDE, boundary, and initial conditions of the wavefield as it optimizes the loss value [23,28]. Although the inclusion of prior knowledge (ICs and BCs) in the training process of NNs to enable prediction of the PDE solution is an approach that has been previously demonstrated in the DL literature [42], PINNs not only include this idea in their framework, but they also advantageously build upon it by utilizing discrete time-stepping while incorporating the physics of the problem in the loss function as they train to predict the PDE solution [23]. Hence, PINNs accomplish a more accurate inference of the PDE solution, while providing a measurement of uncertainty [43]. Following these contributions, the general concept of PINNs was methodically presented in [23] and implemented in [28] to handle the two types of problems, where the governing physics PDE parameters are known. Thus, PINN infers the PDE solution and inverse problems where the solution is known and then uses this to learn the governing physics parameters. In our work, we specifically focused on solving the forward problem of the wave equation.

(5) $$f\left(x,t\right)=\frac{\partial^{2}u}{\partial t^{2}}-c^{2}\left(\frac{\partial^{2}u}{\partial x^{2}}\right)=0$$

To formulate the wave equation (Equation (1)) using PINNs, we rewrite the PDE to comply with the PINNs framework [23]. The input is represented by $x=x_{1},x_{2},x_{3},...,x_{d}$, where d is the highest dimension. Variable $x_{d}$, which is the last vector of dimensions, can also represent the time variable in a multidimensional physical system simulated through a time range expressed by a t vector. Function u(x,t) indicates the solution, and λ is the set of coefficients of the PDE alongside the derivative terms. This initial step creates Equation (5) to be integrated later on in the PINN framework as a loss term to be minimized within the overall loss function.

#### 2.2.1. The PINN Architecture

**Figure 1:**
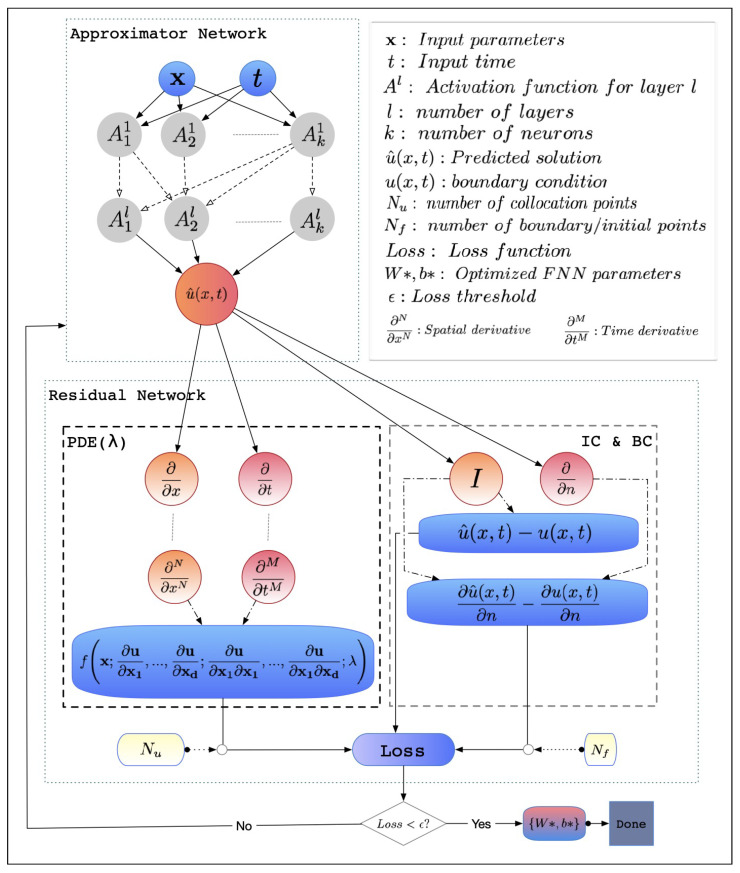
A PINN architecture. The “*” in the figure indicates the updated value of weights and biases. The upper-left neural network represents the approximator network that estimates the solution, $\hat{u}\left(x,t\right)$ of the PDE. The lower residual network forces the PDE, IC, and BC to compute the loss value. The PDE model represents an equation composed of derivatives of different orders.

The baseline PINN architecture consists of two-component networks, as shown in Figure 1, the approximator network and the residual network. These two components are integrated with a feedback mechanism to form PINN dynamics. The approximate network represented in Equation (6) shows the neural network NN composed of *L* layers and (L−1) hidden layers, with $W_{l}$ and $b_{l}$ representing weights and biases, respectively, in every layer *l* to process x, which can be a single-or multidimensional variable. The input and output spaces are $R^{d_{input}}$ and $R^{d_{output}}$ with dimensions $d_{input}$ and $d_{output}$, respectively. The input to the PINN is a set of irregularly sampled domain points, and the output is the predicted result.

(6) $${\mathrm{NN}}^{L}\left(x;W_{l},b_{l}\right):R^{d_{input}}\to R^{d_{output}}$$

Inspired by the universal approximation theorem [44], which indicates that any function can be approximated using a perceptron with only one hidden layer and a finite number of neurons, the approximator network was trained to predict the model result *u* at the collocation point location *x*. This is because of the NN’s ability to approximate complex functions in a compact manner. The approximator NN is composed of neurons and layers through which calculations are performed sequentially. This NN is either shallow, having a single hidden layer, or deep, having two or more hidden layers. Further, this is discussed in Section 3. Both types of NNs can produce an approximation for any continuous function, whether linear or nonlinear [23]. Even with this ability, relying on this feature alone, it is still a challenging task for the neural network to optimize its weights and biases to predict an accurate solution to the PDE; thus, the integration of the second part of the PINNs, the residual network, assists in this process.

The residual network applies the governing physics equation formula to the result of the approximator NN as a part of the loss function. This embodies the characteristic features of PINNs. The task of a residual network is to compute the residual (or loss). Therefore, training is not required [40]. The computed loss is then resupplied to the approximator NN in an iterative design to perform the training process. In the residual network, the derivatives of the PDE were calculated using automatic differentiation (AD) [45]. For instance, if *y* is an equation that can be represented by multiple basic functions such as *A*, *B*, and *C*, as in Equation (7), AD works by applying the chain rule [28] to these basic arithmetic operations to compute derivatives in a form similar to Equation (7).

(7) $$\begin{array}{cc} y & =A\left(B\left(C(x)\right)\right)=A\left(B\left(z_{0}\right)\right)=A\left(z_{1}\right) \end{array}$$

where

$$\begin{array}{cccc} z_{0} & =C(x) & \; & \\ z_{1} & =B(z_{0}) & \; & \\ y & =A(z_{1}). & \; & \end{array}$$

AD can also be referred to as computational differentiation or algorithmic differentiation, and it has already been implemented in machine learning libraries such as TensorFlow [46] and PyTorch [47]. This is a key difference between PINN and conventional methods such as FDM/FEM.

$$\begin{array}{c} \frac{\partial y}{\partial x}=\frac{\partial y}{dz_{1}}\frac{\partial z_{1}}{dz_{0}}\frac{\partial z_{0}}{\partial x}=\frac{\partial A(z_{1})}{\partial z_{1}}\frac{\partial B(z_{0})}{\partial z_{0}}\frac{\partial C(x)}{\partial x} \end{array}$$

As shown in Figure 1, the approximator NN tends to have two or more hidden layers that draw an expression between neurons and activation functions (tanh, ReLU, sin, …, etc.). A commonly used approximator NN is the fully-connected Feed-forward Neural Network (FNN) [28]. Other PINN variations may differ in the design of the approximator network architecture [48,49,50]. For the wave equation, we utilized an FNN as the approximator NN because it is sufficient to solve the majority of PDE problems. It is a general assumption that the larger the NN architecture, the better is the approximation. However, particularly with a limited dataset, a too-large NN may be difficult to train, and a too-small NN may not present an accurate approximation. A different approximator NN setup is required according to the type of problem attempted. This means that certain hyperparameters, such as layers, neurons, learning rate, activation function, and epochs, can be more suitable for some problems than others [23]. Examples of using different approximator NN hyperparameters are discussed in Section 3. It is also possible for the approximator NN to be built from multiple FNNs instead of a single one, as proposed in [51]. Multiple FNNs were proposed in [52] inspired by a finite-basis approach for multiple subdomains.

#### 2.2.2. The Loss Function

PINNs aim to minimize Equation (8), which comprises three main loss terms. The first loss term in Equation (8) shown in Equation (9) represents the offset between any given labeled data and the prediction of the NN. The loss in Equation (10) enforces the defined physical function on the set of points sampled from the geometrical domain by penalizing solutions that do not fit the PDE and, therefore, incorporate the physics of the system in the optimization cycle. The final loss term in Equation (8) shown in Equation (11) represents the initial and boundary conditions that abide by the physics of the defined system. In addition, the $MSE_{b}$ loss term can be a composite of multiple boundary terms, such as the left and right boundaries. The number of collocation points sampled from the domain is considered relatively small compared to the training data required for common NNs. All loss terms utilized the Mean Square Error (MSE) formulation. Optimization in PINNs is a training process that aims to minimize the residual error which, in turn, controls the weights and biases θ=(w,b) of the approximator NN. Loss weights $w_{u}$, $w_{f}$, and $w_{b}$ are used to control the importance of each loss term. The labeled data loss (Equation (9)), PDE loss (Equation (10)), and initial condition and boundary condition loss (Equation (11)) can have different weights. This might be implemented to shift the focus of optimization to one loss term more than the other to fine-tune the accuracy of the PINN prediction. For instance, setting the weight $w_{f}$ in Equation (8) to zero implies that the physical loss is removed from the optimization process, and the PINN is trained without any consideration of the underlying governing equation. Moreover, changing the loss weights can be extended to an adaptive process such as the approach proposed in [53].

(8) $$MSE=w_{u}MSE_{u}+w_{f}MSE_{f}+w_{b}MSE_{b}$$

(9) $$MSE_{u}=\frac{1}{N_{u}}\sum_{i=1}^{N_{u}}||\hat{u}\left(x_{u}^{i},t_{u}^{i}\right)-u^{i}|{|}^{2}$$

(10) $$MSE_{f}=\frac{1}{N_{f}}\sum_{i=1}^{N_{f}}||f\left(\hat{u}\left(x_{u}^{i},t_{u}^{i}\right)\right)-f\left(x_{u}^{i},t_{u}^{i}\right)|{|}^{2}$$

(11) $$MSE_{b}=\frac{1}{N_{b}}\sum_{j=1}^{N_{b}}||f_{b}\left(\hat{u}\left(x_{u}^{j},t_{u}^{j}\right)-f_{b}\left(x_{u}^{j},t_{u}^{j}\right)\right)|{|}^{2}$$

In classical PINNs, loss functions can be optimized using an Adam optimizer followed by a Broyden–Fletcher–Goldfarb–Shanno (BFGS) optimizer [54], which is a quasi-Newtonian, full-batch, gradient-based optimization algorithm [55]. This sequence is a common utilization of both optimizers, as shown in [23], benefiting from their advantages in performance speed and locating the local and global minima. Finally, using the collocation points $N_{f}$, boundary points $N_{b}$, and optional labeled data points $N_{u}$ contributes to the data efficiency of PINNs as a modeling algorithm. We would like to highlight again that when solving the forward problem of the linear wave equation (inference), PINN does not require previous solution data as a training set but instead uses the locations of the collocation points in the defined domain as training points.

The initial and boundary condition implementation, which is the focus of our work, can be either hard or soft constraints. Regardless of the constraint status chosen to implement the ICs/BCs, the number of training points, distribution of data samples, and PINN hyperparameters depend highly on the setup of the PDE problem. This is explored in detail in Section 3. In each modeled problem, predefined ICs and BCs were specified before initiating the PINN training process.

#### 2.2.3. The Activation Functions

Activation functions also play a central role in approximator NNs. They have an apparent effect on the training process. Some commonly used activation functions are the hyperbolic tangent function (tanh), sinusoidal function (sin), sigmoid function, rectified linear unit function (ReLU), and Swish, which is an extension of the Sigmoid-Weighted Linear Unit function (SiLU) [56]. Other activation functions are also used. However, the activation function needs to be smooth to obtain well-defined derivatives for the loss terms, thereby increasing the chances of convergence. Therefore, exponential linear units (ELU) and their scaled versions were avoided. Training speed can also be improved by adding an extra parameter to each hidden layer to modify the slope of the activation function [57]. In the architectures implemented in our experiments, tanh and sin activation functions were used interchangeably according to the status of the constraints tested in that particular case. In the results of the first and last cases, a sin adaptive function is used. The second and third cases utilized an adaptive tanh activation function. These two activation functions were used in our work, as they have shown the most accurate results in the space of our experiments.

#### 2.2.4. The Dynamics of PINNs and the Optimization Process

The residual loss was optimized by changing the weights and biases of the approximator NN to minimize the loss value. This process is the “training” of the network. The resultant value of the loss function is fed back to the approximator NN, where the weights and biases are shifted according to the learning rate, and the PINN undergoes a new iteration of training.

The size of the input data, output data, number of hidden layers, and number of neurons in each layer affect the number of trainable parameters in the FNN. Larger-sized FNNs with larger inputs increase the number of trainable parameters. This large number of trainable parameters requires longer training times to achieve convergence. This makes finding the global minima an NP-hard problem [58].

In our case, training was performed by involving variants of Stochastic Gradient Descent (SGD) methods for error minimization using Adam [59] and BFGS [60]. Training started by applying the Adam optimizer to the constructed model. This training stage was stopped according to the predefined number of training epochs. Subsequently, another stage of training was applied using the L-BFGS-B optimization method. This setup of the optimizer sequence compensates for the limited amount of training data by reducing computational loss and hopefully achieving faster convergence [23,28]. It is also the optimization sequence used to solve all the wave equation problems that were set up in this paper with different combinations of IC and BC constraint statuses.

To combine all the components of PINNs, we summarize the PINN algorithm in a few steps in Algorithm 1. It starts by defining the wave equation problem and its specific ICs/BCs and ends with finding the best PINN parameters (θ) for approximating the wavefield solution.
**Algorithm 1** Physics-informed neural network for solving the wave PDEDefine the training set domain, governing physical formula, and initial/boundary conditions.Initialize the parameters for the approximator network.Compute approximate solution u(x,t).Compute the residual loss by calculating the physical loss and initial and boundary condition losses.Use the residual loss to train the approximator network and optimize its parameters θ (and η if it is also to be inferred) by minimizing the residual loss value.Repeat steps (3–5) until reaching a halt threshold.

### 2.3. Hard and Soft Constraints

The defined initial and boundary conditions for the PDE can be highly informative for PINN training. They can be integrated into PINNs via one of two implementations: soft or hard constraints. The use of soft constraints in PINNs creates the term $MSE_{b}$ in the overall loss function of Equation (8). This tells the PINN to use collocation points located at the borders of the spatial domain and the initial time (or final time if defined) (i.e., if D is the domain of interest, then we choose points at x=min(D), max(D), and/or at t=0). Hence, it validates the initial and boundary conditions and penalizes the PINN predictions that violate the conditions represented in the loss term. This type of implementation of the initial and boundary conditions may not guarantee full satisfaction of these conditions, and the assigned weight $w_{b}$ must have a value proportional to the other weights $w_{u}$ and $w_{f}$ in Equation (8). An affirmative guiding theory for assigning the values of these weights remains an open study [61].

On the other hand, hard constraints can encode the initial or boundary conditions into PINNs by enforcing the approximator NN to satisfy them. This is performed through an output transform function, such as in Equations (13) and (14) via an additional layer of PINNs. Using hard constraints, PINNs have fewer loss terms to optimize but more parameters to train [28,62]. This PINN design can also be referred to as Physics-Constrained Neural Networks (PCNNs) [63].

Modeling the wave equation with PINNs exhibits different performances and accuracies, as we explore the different constraint statuses for ICs and BCs. In Section 3, we focus on the four main cases of implementing the wave equation model in PINNs. In each case, a different constraint status combination is assigned to the ICs and BCs as follows:

- The wave model with hard initial and boundary constraints (hard–hard).
- The wave model with soft initial and boundary constraints (soft–soft).
- The wave model with a hard initial constraint and soft boundary constraints (hard–soft).
- The wave model with a soft initial constraint and hard boundary constraints (soft–hard).

We then present the leading results in performance and accuracy.

## 3. Results and Discussion

A series of PINN models were tested to observe the prediction behavior in multiple PINN setups with different constraint statuses. The PINN models were designed to observe the effect of implementing the initial and boundary conditions in different constraint statuses using either soft, hard, or both constraints interchangeably. While experimenting with each PINN model, we monitored its influence on prediction accuracy. All modeling trials were performed on a machine with an NVIDIA RTX 3090 GPU and Windows operating system. All the designed PINNs were implemented using the Python library DeepXDE [28] with a TensorFlow backend. DeepXDE is a well-known library used for implementing PINNs. Several other tools and libraries can also be used to implement PINNs [64,65,66]. An overview of each library and the differences between them is beyond the scope of this study.

Through the process of model testing, it was noticed that different model setups require a set of different hyperparameters to obtain the best prediction results for that particular model (i.e., the best hyperparameters for obtaining a good prediction while using hard initial and boundary constraints are different from the best hyperparameters to obtain a good prediction while using soft initial and boundary constraints). Therefore, to ensure a fair comparison between the error values, we performed trials of the best-tested hyperparameters of one constraint status combination in all other constraint combinations. This allowed us to observe the prediction accuracy of the set of hyperparameters that worked best for one of the four main cases in the remaining three constraint combinations. The word “trial” here refers to a single run of fully training a PINN model and using it for prediction. The error values reported in the tables in Section 3 are the average values of 10 independent trials for the same randomization seed for each setup while discarding outlier values (unreasonable values that can possibly occur due to initial parameter randomization). The difference in results was noticed to occur even when using the same randomization seed when utilizing the DeepXDE library; hence, the repetition of trials with the same randomization seed was performed. This was performed to reduce the reproducibility of the results when attempting to replicate the experiments in this work.

Each PINN model prediction was compared to an FDM solution obtained previously and treated as the ground truth to measure the solution accuracy. The FDM solution has the same geometry and PDE parameters as those applied to the PINN problem. When performing our trials, we were looking for a model that produces the least an L2 relative error value with an acceptable companion mean residual error (MRE) value, which in turn reflects the PINN’s ability to predict the correct solution of the forward linear wave problem in one dimension.

In our first set of trials, we performed a series of model tests to determine the best hyperparameters for implementing hard initial and boundary constraints (hard–hard). The use of hard constraints for both the initial and boundary conditions removes the initial and boundary loss terms from the overall loss in Equation (8). Because we do not have any additional labeled data, the model loss equation depends solely on the physics loss term ($MSE_{f}$). In other words, Dirichlet initial and boundary conditions are strictly imposed on the prediction of the NN using the output transform function in Equation (12), which is thought of as an additional layer added to the approximator NN.

(12) $$\hat{u}\left(x,t\right)=\left(1-x\right)\left(x\right)\left(t\right)\mathrm{NN}\left(x,t\right)+\left(1-x\right)\sin\left(4\pi ft\right)$$

**Figure 2:**
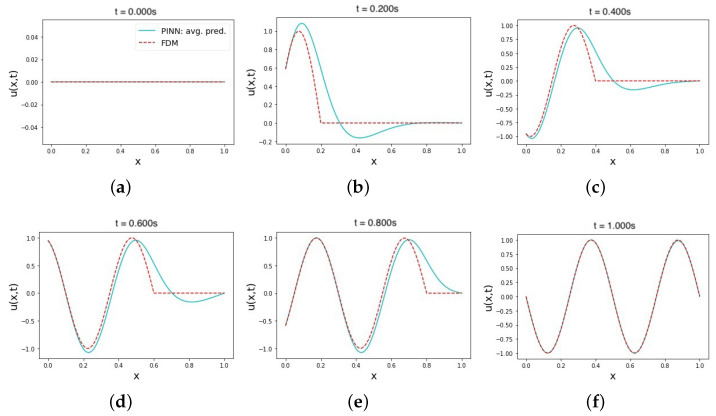
The average solution prediction of the wave equation using a PINN with hard–hard ICs and BCs. Plots (**a**–**f**) show the average of six trials in multiple time instances in the range [0, 1] after removing the four outliers. All training points are sampled uniformly from the domain in all trials.

Setting up PINNs with hard constraints reduces the computational cost by reducing the number of loss terms whose values must be minimized [67]. This impacts the process of fine-tuning the hyperparameters of the PINN, and it requires more testing in a single model trial to find better hyperparameters to enable more precise predictions. As shown in Table 1, the PINN hyperparameters used for obtaining an MRE of 0.38 in the hard–hard constraints setup can obtain lower MRE values using the other constraint statuses of ICs and BCs. This value also hovers around a relatively large offset of 0.4 in comparison with other cases using the same hyperparameters. However, the L2 relative error average value is the lowest ≈0.39 when using this particular set of hyperparameters, yielding the closest prediction to the FDM solution. As shown in Figure 2a,f, the PINN prediction is more accurate than the middle-time instances. This shows the forcing effects of the output transform function in Equation (12), where the prediction output NN(x,t) is enforced to 0 when t=0 or x=1, while it changes the enforced value to sin(4πft) whenever x=0. In the middle-time instances, the enforcement effect is affected by the outcome of the approximator NN NN(x,t). To improve the approximator NN prediction outcome, multiple repeated trials with different hyperparameters are required. Thus far, the results shown are the best of our hyperparameter tests. The PINN model that achieved the best prediction in this case (hard–hard constraints) contained five hidden layers with 100 neurons in each. The learning rate for training was $5\;\times \;10^{-4}$, and the activation function was a non-adaptive sin. This PINN architecture was trained using 50,000 Adam epochs, followed by 10,000 L-BFGS-B epochs. In every epoch, 1600 points were uniformly sampled from the domain and 160 points from the boundary (very close to the boundaries).

**Figure 3:**
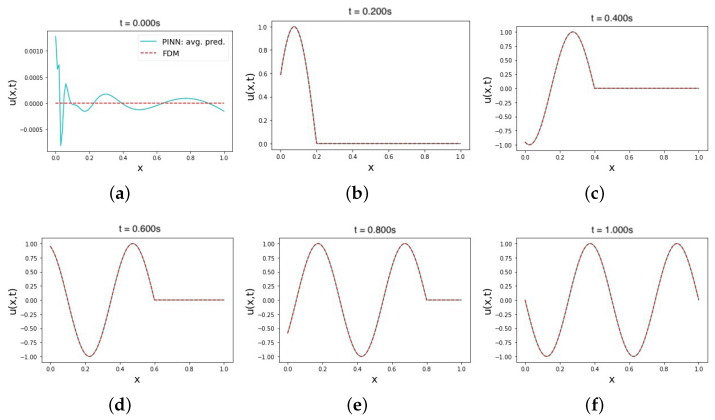
The average solution prediction of the wave equation using a PINN with soft IC and BC constraints (soft–soft). Plots (**a**–**f**) show the average prediction values of 10 trials of multiple time instances in the range [0, 1]. All training points are sampled uniformly from the domain in all trials.

One of the simplest ways to implement IC and BC in PINNs is to treat them as soft model constraints, usually represented by the loss term $MSE_{b}$ in the overall loss function [67]. The use of soft–soft IC and BC constraints in modeling the wave equation is reflected in the results shown in the second row of Table 2. After conducting a series of experiments, the best-performing set of hyperparameters produced a prediction with an MRE of $3.8\;\times \;10^{-4}$ and an L2 relative error of 0.14. These average error values were consistent across the trials, as indicated by the average predicted solution in Figure 3. No output transform function was used in these trials, which may have influenced the shape of the curve in the initial time solution prediction. The prediction output in Figure 3a is not rigidly enforced, as is the case with hard–hard IC and BC constraints, but is instead approximated to best meet the soft IC, starting from the Glorot normal initialization [68] to the NN trainable parameters in the approximator. During the testing trials, the set of hyperparameters used to train a soft–soft constraint model performed well, despite minor fluctuations in the solution prediction values at the initial time. Although the same set of hyperparameters was used with other combinations of IC and BC constraints, as shown in the rest of the rows of Table 2, their predictions were not as accurate. This confirms that applying soft–soft constraints in modeling the wave equation with PINNs requires a different set of hyperparameters than those needed to achieve the best prediction results with other constraint statuses. Additionally, fine-tuning the loss weights in the loss function was easier, given the clear impact of changes on the convergence of the training error. The PINN architecture that achieved this accurate prediction with soft–soft constraints consisted of six hidden NN layers, with the first two layers consisting of 128 neurons, and the remaining four layers consisting of 64 neurons. The PINN was trained with a learning rate of $1\;\times \;10^{-3}$, which decreased over 2000 iterations out of the total 10,000 Adam training epochs. The activation function used was an adaptive tanh with a slope factor of five. This was followed by 10,000 additional L-BFGS-B training epochs. The physics, initial, and left boundary loss terms were assigned a higher weight in the loss function to ensure equal minimization momentum across all loss terms contributing to the overall loss equation.

We also conducted trials with different combinations of initial and boundary conditions (ICs and BCs) in the same PINN model, using either hard ICs and soft BCs or vice versa, as shown in Table 3 and Table 4. In these two sets of trials, the output transformation functions (Equations (13) and (14)) were applied to the NN predictions in the hard–soft and soft–hard cases, respectively. Equation (13) was used to enforce a hard initial constraint in the hard–soft model, while Equation (14) was applied to the soft–hard model to impose hard boundary constraints. It is important to note that the main difference between Equations (12) and (14) is the presence of *t* in the first term of the equation. The presence of *t* in Equations (12) and (13) is used to control the ICs. For example, when *t* equals zero, the prediction is neglected and forced to zero as well.

(13) $$\hat{u}\left(x,t\right)=\left(t\right)\mathrm{NN}\left(x,t\right)$$

(14) $$\hat{u}\left(x,t\right)=\left(1-x\right)\left(x\right)\mathrm{NN}\left(x,t\right)+\left(1-x\right)\sin\left(4\pi ft\right)$$

**Figure 4:**
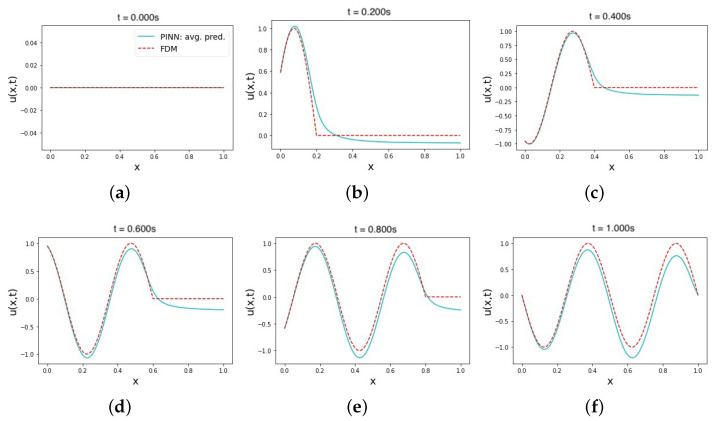
The average predicted solution of the wave equation using PINN with hard IC constraints and soft BCs constraints (hard–soft). Plots (**a**–**f**) show the average prediction values of the four most accurate trials in multiple time instances in the range [0, 1]. The six remaining trials that showed an L2 relative error ≈> 1.0 were excluded. All training points are sampled uniformly from the domain in all trials.

In Table 3, the average error values of using the best-tested set of hyperparameters in the hard IC constraint and soft BC (hard–soft) constraint model are shown. The trials show a stable average MRE of 0.018 and L2 relative error of 0.27 ± 0.11. To acquire these error values, the PINN model was constructed from seven hidden NN layers, where the first two layers had 256 neurons, and the remaining five layers had 64 neurons each. The learning rate was assigned to $1\;\times \;10^{-3}$, and an inverse time decay was applied over the 10,000 Adam training epochs. The activation function used is an adaptive tanh with a value of two for the slope factor. This is followed by 10,000 L-BFGS-B training epochs. The training was performed over 1600 domain points and 160 boundary points uniformly sampled. We observed that assigning higher weights for the physics ($MSE_{f}$) and left boundary conditions in the total loss equation produced better prediction values. We assume a potential reason for this occurrence is that learning a function requires more emphasis than learning a constant value in PINNs. The same set of hyperparameters performed very poorly in terms of the MRE for the hard–hard constraints model, as shown in the first row in Table 3. While these are the best-tested hyperparameters for this case, applying them to the soft–soft constraints model reflects lower error values that translate to better performance. The solution prediction results for this trial set are summarized in Figure 4. Using hard constraints for the initial time in Figure 4a enforces a zero value to all prediction values at that time instance. However, the prediction results in the rest of the time instances in Figure 4b–f become less accurate than in the soft–soft case. This occurrence is possibly the result of omitting the initial condition loss term from the loss function and instead relying on enforcing it through the output transform function. This leaves the approximator NN training process dependent on minimizing the physics ($MSE_{f}$) and BC loss ($MSE_{b}$) values alone instead of considering the IC loss value as well.

**Figure 5:**
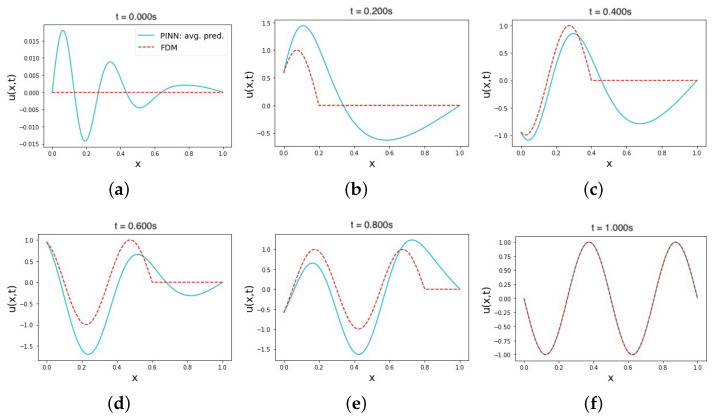
The average predicted solution of the wave equation using a PINN with soft initial constraints and hard boundary constraints (soft–hard). Plots (**a**–**f**) show the average prediction values of the five most accurate trials of multiple time instances in the range [0, 1]. The rest of the trials that reflected an L2 relative error > 1.0 were excluded. All training points are sampled uniformly from the domain in all trials.

In Table 4, a trial set is executed to identify the performance accuracy of the soft–hard IC and BC constraint PINN model using the best-tested hyperparameters for the case. As computed in the fourth row of Table 4, the best-tested hyperparameters for this case reflected an average MRE value of 0.018, and an L2 relative error of 0.87 ± 0.07 over the performed training and prediction trials. The PINN setup used for this case is composed of five hidden NN layers containing 64 neurons each. The learning rate used is $4\;\times \;10^{-4}$, and the activation function was a non-adaptive sin. The PINN was trained through 12,000 Adam epochs followed by 10,000 L-BFGS-B epochs. Maintaining the same set of hyperparameters and changing the IC and BC constraint status shows the error results summarized in Table 4. Despite showing reasonable results for modeling the wave equation with soft–hard IC and BC constraints, these PINN hyperparameters performed with a degraded MRE value for hard IC and BC constraints. They also show a poor L2 relative error value when applying them to a soft–soft IC and BC constraint model setup. Nevertheless, the best-tested hyperparameters for the soft–hard constraints perform better when switching status to hard–soft constraints. The plots in Figure 5 display the average prediction results for the soft–hard constraints model relative to the FDM solution. The enforcing effect of applying hard BC constraints is clearly visible in the last time instance in Figure 5f. The small-valued disruptions shown in Figure 5a reflect the PINN prediction of the soft IC in the initial time instance. The average solution prediction of the middle time instances in Figure 5b–e is the outcome of training PINNs with the best-tested set of hyperparameters for the soft–hard constraints combination. At its best-tested performance, this case does not show a better average prediction than the soft–soft constraints model.

The highlights of the thoroughly studied four cases for IC and BC constraints in this work reveal important behaviors when attempting to model the one-dimensional wave forward problem. Using soft constraints allows for flexibility in composing the governing ICs and BCs in the domain of interest while still enabling control over loss weights as each of the physics, initial, and boundary loss terms are defined independently in the total loss equation. The trial sets show that a soft–soft constraints model achieved the lowest L2 relative error value. Choosing the optimal hyperparameters for PINNs is highly dependent on the problem design and the available computational resources. The problem design here includes the choice of constraint status chosen to implement the IC and BCs of the system.

As for the time performance of each of the constraint statuses, Table 5 shows the training and prediction times for a single PINN model trial of each of the four studied constraint combinations with their most appropriate set of hyperparameters. Regardless of the status of the constraints chosen during training the PINN, the prediction times remain consistent at approximately 0.4 s. However, the training time consumed for a PINN model with hard–soft constraints is the greatest, while the least is in the training of a PINN model with soft–hard constraints. This can be explained by the approximator NN size along with its suitable set of hyperparameters that are used to achieve the results reported in Section 3. The training times do not necessarily reflect a better performance of one over another because of factors like the size of the PINN. The number of neurons and layers alone can play a main role in increasing or decreasing the training time. Considering the difference of the most suitable set of hyperparameters adds to the reason for the training time difference among the studied cases of this work. For solving the 1D wave equation problem, Table 5 puts on view the FDM solution time of 0.01 s, which ranks the fastest among the prediction times. This is particular to the 1D case. However, it is expected in higher dimensional problems that the FDM consumes more time exponentially given its meshed-based origin. This is not the case in PINN models. The increase in dimensions is not reported to suffer from such an increase in prediction time [31].

## 4. Conclusions

In this work, we implemented ICs and BCs with a focus on the wave equation problem with a time-dependent source term in four possible combinations of soft and hard constraints. By staging these findings, we show that a continuous time-dependent source point can be accurately modeled using a combination of soft–soft constraints for implementing IC and BC, demonstrating an L2 relative error of 0.14. This prediction error value is approximately 63%, 48%, and 83% lower than those of other hard–hard, hard–soft, and soft–hard combinations, respectively, with their best-explored hyperparameters. In addition, we demonstrated that the flexibility of using soft constraint combinations in PINNs permits the addition of the required physical relations as additional terms in the loss equation with appropriate loss weights. The study of constraint modeling cases performed in our work is a step toward the easier adoption of PINNs as a mesh-free efficient modeling method in ultrasound therapeutics and safe noninvasive surgery.

In a future study, we aim to explore other architectures of PINNs such as the Spatio-Temporal Multi-scale Fourier Neural Networks (STMsFNNs), which target problems with a higher frequency solution tendency or multi-scale parameters. We also aim to extend the research on using multi-point time-dependent source functions in multi-dimensional wavefields in homogeneous and inhomogeneous media.

## Figures and Tables

**Table 1:** Error evaluation of using the best experimental hyperparameters for hard initial and boundary constraints (hard–hard) model.

Constraint Status	Mean Residual Error		L2 Relative Error	
	**Avg.**	**Std. Dev.**	**Avg.**	**Std. Dev.**
**Hard IC Hard BC**	**0.3837269**	**0.4074091**	**0.3897162**	**0.0478062**
Soft IC Soft BC	0.0162678	0.0102160	4.2226975	3.4472078
Hard IC Soft BC	0.0010502	0.0001981	0.7667636	0.1176193
Soft IC Hard BC	0.0090117	0.0009739	0.8214965	0.1732684

**Table 2:** Error evaluation of using the best-tested hyperparameters for soft IC and BC constraints (soft–soft) model.

Constraint Status	Mean Residual Error		L2 Relative Error	
	**Avg.**	**Std. Dev.**	**Avg.**	**Std. Dev.**
Hard IC Hard BC	1.5917058	1.3165160	1.5262532	0.6745228
**Soft IC Soft BC**	**0.0003816**	$5.5110\;\times \;10^{-5}$	**0.1400467**	$2.3701\;\times \;10^{-5}$
Hard IC Soft BC	0.0041027	0.0008690	0.9136354	0.1429870
Soft IC Hard BC	0.0304965	0.0105405	1.3186286	0.3858241

**Table 3:** Error evaluation of using the best-tested set of hyperparameters for a hard IC constraint and soft BC constraint (hard–soft) model.

Constraint Status	Mean Residual Error		L2 Relative Error	
	**Avg.**	**Std. Dev.**	**Avg.**	**Std. Dev.**
Hard IC Hard BC	6.3646400	7.0906469	1.4607935	0.3224401
Soft IC Soft BC	0.0036384	0.0069507	0.2422296	0.2472808
**Hard IC Soft BC**	**0.0185242**	**0.0080893**	**0.2743762**	**0.1135189**
Soft IC Hard BC	0.0612217	0.0153550	1.3467069	0.3455660

**Table 4:** Error evaluation of using the best-tested set of hyperparameters in a soft IC constraint and hard BC constraint (soft–hard) model.

Constraint Status	Mean Residual Error		L2 Relative Error	
	**Avg.**	**Std. Dev.**	**Avg.**	**Std. Dev.**
Hard IC Hard BC	3.9619709	0.6002788	0.7098207	0.0747314
Soft IC Soft BC	0.0279267	0.0181732	12.600045	5.6582992
Hard IC Soft BC	0.0017451	0.0003707	0.7805654	0.1108798
**Soft IC Hard BC**	**0.0187903**	**0.0019859**	**0.8758570**	**0.0733874**

**Table 5:** Execution times for each of the constraint statuses with their best-tested set of hyperparameters.

Constraint Status	Training Time	Prediction Time
Hard IC Hard BC	65.04 min	0.42 s
Hard IC Soft BC	84.47 min	0.48 s
Soft IC Hard BC	33.61 min	0.45 s
Soft IC Soft BC	41.31 min	0.49 s
FDM	-	0.01 s

## Data Availability

The data presented in this study are available on request from the corresponding author. The data are not publicly available due to privacy and ongoing research on higher dimensions of the problem.

## Abbreviations

The following abbreviations are used in this manuscript:

PDE	Partial differential equation
DNN	Deep neural network
IC	Initial condition
BC	Boundary condition
PINN	Physics-informed neural network
FDM	Finite difference method
FEM	Finite element method
